# A pathway to improved maternity research in Northern Ireland? Novel linkage of Northern Ireland Longitudinal Study and Northern Ireland Maternity Services System

**DOI:** 10.23889/ijpds.v9i5.1645

**Published:** 2024-09-18

**Authors:** Estelle Lowry, Neil Rowland

**Affiliations:** 1 Queen's University Belfast

## Objective

Our primary objective was to determine the social and demographic determinants of interpregnancy weight change. A secondary aim is to highlight the novel linkage of two data resources in Northern Ireland (NI) and the potential for transformative research in maternal health.

## Approach

We piloted a process to link two datasets in a safe, legal and ethical manner. The NI Maternity Services System (NIMATS) provides access to biological variables collected during the gestational period. The NI Longitudinal Study (NILS) is a rich source of Census information providing a demographic, social and economic background we would not otherwise obtain from NIMATS alone.

## Results

We piloted a process to link two datasets in a safe, legal and ethical manner. The NI Maternity Services System (NIMATS) provides access to biological variables collected during the gestational period. The NI Longitudinal Study (NILS) is a rich source of Census information providing a demographic, social and economic background we would not otherwise obtain from NIMATS alone.

## Conclusion & Implications

The neuroimaging ingest pipeline developed has allowed for the distribution of imaging datasets within DPUK which has facilitated multi-modal research on anonymised and standardised data and enabled linkage with phenotypic and genomic data.

